# Prevalence of Overweight and Obesity and Weight Loss Practice among Beijing Adults, 2011

**DOI:** 10.1371/journal.pone.0098744

**Published:** 2014-09-16

**Authors:** Li Cai, Xiaoyan Han, Zhi Qi, Zhe Li, Yumei Zhang, Peiyu Wang, Aiping Liu

**Affiliations:** 1 Department of Nutrition and Food Hygiene, School of Public Health, Peking University Health Science Center, Beijing, China; 2 Chaoyang District Centre for Disease Control and Prevention, Beijing, China; 3 Department of Social Medicine and Health Education, School of Public Health, Peking University Health Science Center, Beijing, China; 4 School of Public Health, Sun Yat-sen University, Guangzhou, China; CUNY, United States of America

## Abstract

**Objective:**

This study aims to determine the up-to-date prevalence of overweight and obesity, the distributions of body weight perception and weight loss practice in Beijing adults.

**Methods:**

A cross-sectional study was conducted in 2011. A total of 2563 men and 4088 women aged 18–79 years from the general population were included. Data were obtained from questionnaire and physical examination.

**Results:**

The prevalence of overweight (BMI 24–27.9 kg/m^2^) and obesity (BMI≥28 kg/m^2^) was 42.1% and 20.3% in men and 35.6% and 17.1% in women, respectively. Age was inversely associated with overweight in both sexes, and obesity in women. Education level was negatively associated with overweight and obesity in women but not in men. Only 49.1% men and 58.3% women had a correct perception of their body weight. Underestimation of body weight was more common than overestimation, especially in men, the older people, and those with low education level. The percentage of taking action to lose weight was inversely associated with men and old age, and positively associated with higher education level, higher BMI, and self-perception as “fat” (OR = 3.78 in men, OR = 2.91 in women). Only 26.1% of overweight/obese individuals took action to lose weight. The top two weight loss practices were to reduce the amount of food intake and exercise.

**Conclusion:**

Overweight and obesity were highly prevalent with high incorrect body weight perceptions in the general adult population in Beijing. Weight loss practice was poor in overweight and obese individuals. Actions at multiple levels are needed to slow or control this overweight and obesity epidemic.

## Introduction

Overweight and obesity are highly prevalent in western countries and growing problems in developing countries. The prevalence of overweight and obesity in China was relatively low compared with western countries but has increased rapidly during the past decades [1]–[3], especially in large cities. National survey showed that the prevalence of overweight and obesity increased over 40% in Chinese adults aged 18–59 years from 1992 to 2002 [3]. In 2002, 22.8% of the adult population were overweight (body mass index (BMI): 24–27.9 kg/m^2^) and another 7.1% were obese (BMI≥28 kg/m^2^) [4]. The prevalence of overweight and obesity in Chinese adults was as high as 39.6% in 2009 [1]. There was no significant increase in the prevalence of obesity in the United States over the past 10 years [5]. Researchers also observed a decrease in the prevalence of overweight (BMI≥25 kg/m^2^) in Japanese women during 1985 to 2005 [6]. However, the observed increasing trend in the prevalence of overweight and obesity in China is predicted to continue in the coming years [7]. An estimation of the up-to-date prevalence of overweight and obesity is essential to understand this epidemic.

Overweight and obesity have been shown to be related to various cardiovascular disease risk factors and increased cardiovascular disease events [8]–[10]. Weight loss and weight control are therefore important considerations for public health benefit. It has been demonstrated that body weight perception was strongly associated with weight control practice [11]–[14]. Overweight people tend to underestimate their body weight [15], which may contribute to the weight increase/maintenance. Appropriate body weight perception was estimated to be an important point of focus for the design and implementation of clinical and public health initiatives [16]. However, little is known about the weight perceptions and weight loss practices in Chinese adults.

Beijing, the capital of China, is one of the large cities that have the highest prevalence of overweight and obesity. Therefore, using population estimates, this study aims to provide the up-to-date prevalence of overweight and obesity and investigate weight perception and weight loss practice among Beijing adults.

## Materials and Methods

### Ethics Statement

Ethical approval was given by the Ethics Committee of Beijing Centers for Disease Control and Prevention. Written informed consent was obtained from all participants before the enrollment.

### Study population

Data used were from the 2011 survey of cardiovascular risk factors in Beijing adult population aged 18 to 79 years. We used a stratified multistage probability proportional to size sampling design. The first stage involved a random selection of 23 streets/townships. The second stage involved a random selection of one residential committee/village and two organizations from each selected streets/townships. In the third stage, one group consisting of approximately 100 households was randomly sampled from each selected residential committees/villages and organizations. In the fourth stage, one individual from each designated households was chosen using a Kish table. When the selected individual refused or was unavailable, a replacement household was selected from the same neighborhood/village. We ensured a sufficient sample size for estimating the prevalence of overweight and obesity in men and women, respectively. For example, the required sample size was 1537 for men and 1750 for women (based on parameters as following: prevalence of obesity = 20% for men and 18% for women, α = 0.05 and δ = 0.02 for men and 0.018 for women). Finally, 6917 people participated the study, 266 participants with missing information on sex, age, and/or weight status were excluded, and 6651 (2563 men and 4088 women) were included in the analysis.

### Data collection

#### Questionnaire interview

Data collection was performed in physical examination centers at local health stations or community clinics in the participants' residential area. A standardized questionnaire was administered by trained research staffs to obtain information on demographics and other covariates.

Body weight perceptions were determined by the following questions. Participants were asked, “How do you describe your body weight?” The answer categories included: (1) too thin; (2) a little thin; (3) normal; (4) a little fat; (5) too fat; (6) unknown (i.e., have no concept of their body weight or have not paid attention to that). However, for analysis, “too thin” and “a little thin” were merged to form a “thin” group and “a little fat” and “too fat” were merged to form a “fat” group.

Weight loss status and practices were determined by the following questions. Participants were asked, “During the past 12 months, have you taken any action to lose weight?” Those answered “yes” were asked, “Which practice(s) have you adopted to lose weight?” The answer categories included: (1) reduce the amount of food intake; (2) low-fat diet; (3) low-calorie diet; (4) exercise; (5) use drug treatment; and (6) others. Respondents were allowed to select as many as were applicable.

#### Anthropometric measurement

Standing height, weight and waist circumferences were measured by research staffs during the interview. Height was measured without shoes and hat in an upright position with a vertical height gauge to the nearest 0.1 cm. Weight was measured using an electronic scale to the nearest 0.1 kg after removal of shoes, hat, heavier clothing and pocket contents. BMI (kg/m^2^) was calculated as weight in kilograms divided by height in meters squared. Waist circumference was measured half way between the lowest rib margin and the iliac crest at the end of a normal expiration.

### Definitions

The Chinese BMI classifications: underweight (BMI<18.5 kg/m^2^), normal weight (BMI 18.5–23.9 kg/m^2^), overweight (BMI 24–27.9 kg/m^2^), and obesity (BMI≥28 kg/m^2^).

Participants were considered to have a correct weight perception if they met one of the following criteria: (1) underweight (BMI<18.5 kg/m^2^) and perceived themselves as “thin”; (2) normal weight (BMI 18.5–23.9 kg/m^2^) and perceived themselves as “normal”; (3) overweight or obese (BMI≥24 kg/m^2^) and perceived themselves as “fat”. Those had an under, over, or “unknown” perception were considered to have an incorrect weight perception.

### Statistic analysis

All the analyses were conducted using SAS software (version 9.2; SAS Institute, Cary, NC). Two-tailed P<0.05 was considered to be statistically significant. The differences in proportions of categorical variables were determined using χ^2^ test (for sex and perception of body weight), or trend χ^2^ test (for age, education and BMI category).

The prevalence of overweight and obesity was calculated by sex, age, and education level according to both the Chinese and the WHO BMI classifications. The corresponding adjusted odds ratios (ORs) and 95% confidence intervals (CI) for the prevalence of overweight, and obesity were determined by a multiple logistic regression model. The regression model included age and education level as independent variables and was stratified by sex. A nonstratified version of the model was also performed to assess the main effect of sex.

The percentage distributions of body weight perception (thin, normal, fat and unknown) were calculated by sex and BMI. The percentage of correct and incorrect (under, over, or unknown) weight perception were calculated by sex, age, education level, and BMI. The percentage of taking action to lose weight among all participants was calculated by sex, age, education, BMI, and weight perception. The corresponding adjusted ORs and 95% CI were determined by a multiple logistic regression model. Similarly, a stratified (by sex) version and a nonstratified version of model were both performed. Independent variables in the stratified model included age, education level, BMI, and body weight perception. In addition, the percentage of taking action to lose weight among overweight or obese participants were compared between those having appropriate weight perception and those having inappropriate weight perception by sex and age. The percentage of specific weight loss practice among all the persons taking action to lose weight was compared between men and women. The corresponding percentage among overweight or obese persons was also calculated.

## Results

A total of 2563 men and 4088 women were included in the analysis. The mean age of men (47.3±15.1 years) and women (47.2±15.1 years) was similar. Men had a slightly higher mean BMI than women (25.3±3.8 kg/m^2^ vs. 24.5±3.9 kg/m^2^). The mean waist circumference was 86.5±10.4 cm for men and 79.8±10.3 cm for women. A large proportion of men (42.4%) had an education level lower than high school, followed by high school (33.9%), and college or above (23.7%); while the corresponding proportion in women was 39.4%, 31.8% and 28.8%, respectively.

### Prevalence of overweight and obesity

According to the Chinese BMI classification, the prevalence of overweight and obesity was 38.1% and 18.3% among adults in Beijing, respectively (**Table 1**). Men had significantly greater odds of being overweight (OR = 1.32) and obesity (OR = 1.23) compared to women. For men, the prevalence of overweight increased with age, peaking at 50–59 years old; such age trend was not seen in the prevalence of obesity. For women, both the prevalence of overweight and obesity increased with age, peaking at 60–79 years old. The prevalence of overweight and obesity decreased with education level in women but not in men. After adjusted for age, women with an education level of college or above had lowest odds of being overweight (OR = 0.78) or obese (OR = 0.47) compared to those with an education level lower than high school.

**Table 1:** Prevalence of overweight and obesity by selected characteristics and the corresponding odds ratios in Beijing Adults aged 18–79 years, 2011§.

	*n* (%)				Odds Ratios*			
	Men (*n* = 2563)		Women (*n* = 4088)		Men		Women	
	Overweight	Obesity	Overweight	Obesity	Overweight	Obesity	Overweight	Obesity
**Total**	1080 (42.1)†	521 (20.3)†	1455 (35.6)	699 (17.1)	1.32 (1.19, 1.47)	1.23 (1.08, 1.39)	1.00	1.00
**Age**								
18–29	128 (29.8)†	68 (15.9)†	111 (16.7)	34 (5.1)	1.00	1.00	1.00	1.00
30–39	153 (37.4)†	101 (24.7)†	205 (28.2)	85 (11.7)	1.41 (1.06, 1.88)	1.74 (1.23, 2.45)	1.91 (1.47, 2.48)	2.26 (1.50, 3.43)
40–49	255 (42.4)	128 (21.3)	385 (40.5)	187 (19.7)	1.73 (1.33, 2.25)	1.41 (1.02, 1.95)	3.17 (2.48, 4.06)	3.66 (2.49, 5.39)
50–59	290 (48.7)‡	119 (20.0)	350 (42.1)	172 (20.7)	2.21 (1.70, 2.88)	1.32 (0.95, 1.84)	3.30 (2.56, 4.25)	3.82 (2.58, 5.66)
60–79	254 (48.0)	105 (19.9)	404 (44.2)	221 (24.2)	2.21 (1.69, 2.90)	1.28 (0.92, 1.80)	3.56 (2.76, 4.59)	4.22 (2.86, 6.24)
*P* for trend	<0.001	0.627	<0.001	<0.001				
**Education**								
<High school	455 (41.9)	232 (21.4)	658 (41.0)	387 (24.1)	1.00	1.00	1.00	1.00
High school	389 (44.9)†	165 (19.1)‡	468 (36.1)	200 (15.4)	1.18 (0.98, 1.42)	0.87 (0.69, 1.09)	0.98 (0.84, 1.15)	0.69 (0.56, 0.83)
≥College	235 (38.7)†	122 (20.1)†	321 (27.4)	111 (9.5)	0.98 (0.79, 1.20)	0.92 (0.72, 1.18)	0.78 (0.65, 0.93)	0.47 (0.37, 0.60)
*P* for trend	0.353	0.419	<0.001	<0.001				

*Odds ratios were adjusted for age, and education level.

† *P*<0.01: men vs. women.

‡ *P*<0.05: men vs. women.

§ Body size was categorized according to the Chinese criteria for defining overweight (BMI 24–27.9) and obesity (BMI≥28).

According to the WHO BMI classification, the prevalence of overweight (BMI 25–29.9 kg/m^2^) and obesity (BMI≥30 kg/m^2^) was 36.8% (41.3% in men, 34.0% in women) and 8.4% (9.4% in men, 7.9% in women), respectively. The relationships between prevalence of overweight/obesity and sex, age, and education level were not changed when applying the WHO BMI classification (**Table S1**).

### Body weight perceptions

As shown in **Table 2**, with respect to self-reported body weight perception, most men were in the “normal” category (41.6%), while most women were in the “fat” category (47.6%). A substantial percentage of men and women misclassified their own body weight relative to the Chinese BMI classifications. Discrepancies of particular note include the fact that 14.5% of normal weight men considered themselves “fat,” while 49.3% of overweight men considered themselves “normal” or “thin”; the corresponding percentages in women were 32.0% and 37.6%.

**Table 2:** Percentage distributions of body weight perceptions by sex and BMI*.

	Men (%)				Women (%)			
	Thin	Normal	Fat	Unknown	Thin	Normal	Fat	Unknown
**All**	14.2†	41.6	36.1†	8.2†	7.5	40.0	47.6	4.9
**BMI**								
<18.5	60.4	26.4	5.7	7.6	47.7	36.2	11.4	4.7
18.5–23.9	28.0†	48.2†	14.5†	9.3†	9.1	53.5	32.0	5.4
24–27.9	5.0	44.3†	42.8†	8.0†	3.6	34.0	57.8	4.6
≥28	4.6	25.9†	62.8†	6.7	3.0	18.6	73.9	4.4
*P* for trend	<0.001	<0.001	<0.001	0.119	<0.001	<0.001	<0.001	0.319

*Perceptions of body weight were categorized into four groups: thin, normal, fat, and unknown.

† *P*<0.01: men vs. women.

^‡^ *P*<0.05: men vs. women.

**Table 3** displays the percentage of body weight perceptions. Only 49.1% men and 58.3% women had a correct perception of their body weight. Higher percentage of correct perception was related to women, higher education level, and higher BMI. Among those overweight and obese, 49.3% men and 63.0% women had a correct perception. Underestimation was more common than overestimation, especially in men, the older people, and those with a low education level. Over perception was most prevalent in young women. Notably, 8.2% men and 4.9% women reported that they had no concept of their body weight or had not paid attention to that.

**Table 3:** Percentage of body weight perceptions by selected characteristics*.

	Men					Women				
		Percentage					Percentage			
	n	Under perception	Correct perception	Over perception	Unknown	n	Under perception	Correct perception	Over perception	Unknown
**All**	2562	36.9†	49.1†	5.8†	8.2†	4083	21.1	58.3	15.7	4.9
**Age**										
18–29	429	30.5†	52.7	7.9†	8.9†	662	8.9	48.8	37.6	4.7
30–39	409	32.3†	55.0‡	4.7†	8.1	726	10.7	62.3	21.5	5.5
40–49	601	38.8†	45.9†	6.5†	8.8	950	18.7	62.3	12.7	6.2
50–59	595	34.6†	50.6†	5.9‡	8.9†	831	24.6	62.6	8.7	4.2
60–79	528	46.0†	43.8†	4.2	6.1	914	37.3	54.1	4.7	3.9
*P* for trend		<0.001	0.003	0.061	0.218		<0.001	0.207	<0.001	0.190
**Education**										
<High school	1084	41.2†	45.7†	6.3‡	6.8	1605	30.9	55.1	8.9	5.1
High school	866	37.3†	48.2†	4.4†	10.2†	1294	17.7	61.9	16.2	4.3
≥College	607	28.8†	56.5	7.1†	7.6‡	1170	11.0	59.1	24.7	5.2
*P* for trend		<0.001	<0.001	0.766	0.324		<0.001	0.018	<0.001	0.988
**BMI**										
<18.5	53	0.0	60.4	32.1‡	7.6	149	0.0	47.7	47.7	4.7
18.5–23.9	908	28.0†	48.2†	14.5†	9.3†	1781	9.1	53.5	32.0	5.4
24–27.9	1080	49.3†	42.8†	0.0	8.0†	1455	37.6	57.8	0.0	4.6
≥28	521	30.5†	62.8†	0.0	6.7	698	21.6	73.9	0.0	4.4
*P* for trend		<0.001	<0.001	<0.001	0.119		<0.001	<0.001	<0.001	0.319

*Perceptions of body weight were categorized into four groups: under, correct, over, and unknown perception.

† *P*<0.01: men vs. women.

‡ *P*<0.05: men vs. women.

### Percentage of taking action to lose weight

The percentage of taking action to lose weight was lower in men (18.3%) than in women (25.2%) and decreased with age in both sexes (**Table 4**). This percentage increased with education level and BMI in both men and women, respectively. Those perceiving themselves as “fat” were more likely to take action to lose weight compared to their counterparts. Results from the multiple logistic regression models revealed that the percentage of taking action to lose weight was inversely associated with men, old age (60–79 years in men, 50–79 years in women), and self-perceiving as “thin” and positively associated with higher education level (≥college in men, ≥high school in women), higher BMI (≥28 in men, 24–27.9 in women), and self-perception as “fat”. Self-perception as “fat” had the highest OR for taking action to lose weight in both sexes (OR = 3.78 in men, OR = 2.91 in women).

**Table 4:** Percentage of taking action to lose weight by selected characteristic and the corresponding odds ratios.

	Percentage		OR*	
	Men	Women	Men	Women
**All**	18.3†	25.2	0.77 (0.67, 0.88)	1.00
**Age**				
18–29	19.0†	30.5	1.00	1.00
30–39	22.3†	30.5	1.05 (0.73, 1.50)	0.97 (0.76, 1.25)
40–49	18.0†	26.8	0.93 (0.66, 1.31)	0.86 (0.67, 1.10)
50–59	20.0	22.8	0.98 (0.70, 1.38)	0.75 (0.58, 0.98)
60–79	13.0‡	17.8	0.66 (0.45, 0.96)	0.67 (0.51, 0.89)
*P* for trend	0.012	<0.001		
**Education**				
<High school	16.5	18.1	1.00	1.00
High school	16.8†	26.4	0.99 (0.77, 1.28)	1.48 (1.23, 1.80)
≥College	23.6†	33.8	1.38 (1.06, 1.81)	2.15 (1.76, 2.63)
*P* for trend	0.001	<0.001		
**BMI**				
<18.5	1.9‡	14.9	0.23 (0.03, 1.73)	0.8 (0.48, 1.32)
18.5–23.9	11.2†	22.2	1.00	1.00
24–27.9	20.3†	28.0	1.25 (0.94, 1.66)	1.23 (1.02, 1.48)
≥28	28.0	29.5	1.50 (1.09, 2.07)	1.24 (0.98, 1.57)
*P* for trend	<0.001	<0.001		
**Perception**				
Thin	5.6	8.6	0.55 (0.33, 0.91)	0.54 (0.35, 0.83)
Normal	10.6†	15.6	1.00	1.00
Fat	34.6	37.6	3.78 (2.93, 4.87)	2.91 (2.44, 3.48)
Unknown	7.7	8.0	0.67 (0.39, 1.17)	0.44 (0.26, 0.75)
*P* value	<0.001	<0.001		

*Odds ratios were adjusted for sex, age, education level, BMI, and perception of body weight.

† *P*<0.01: men vs. women.

‡ *P*<0.05: men vs. women.

**Figure 1:**
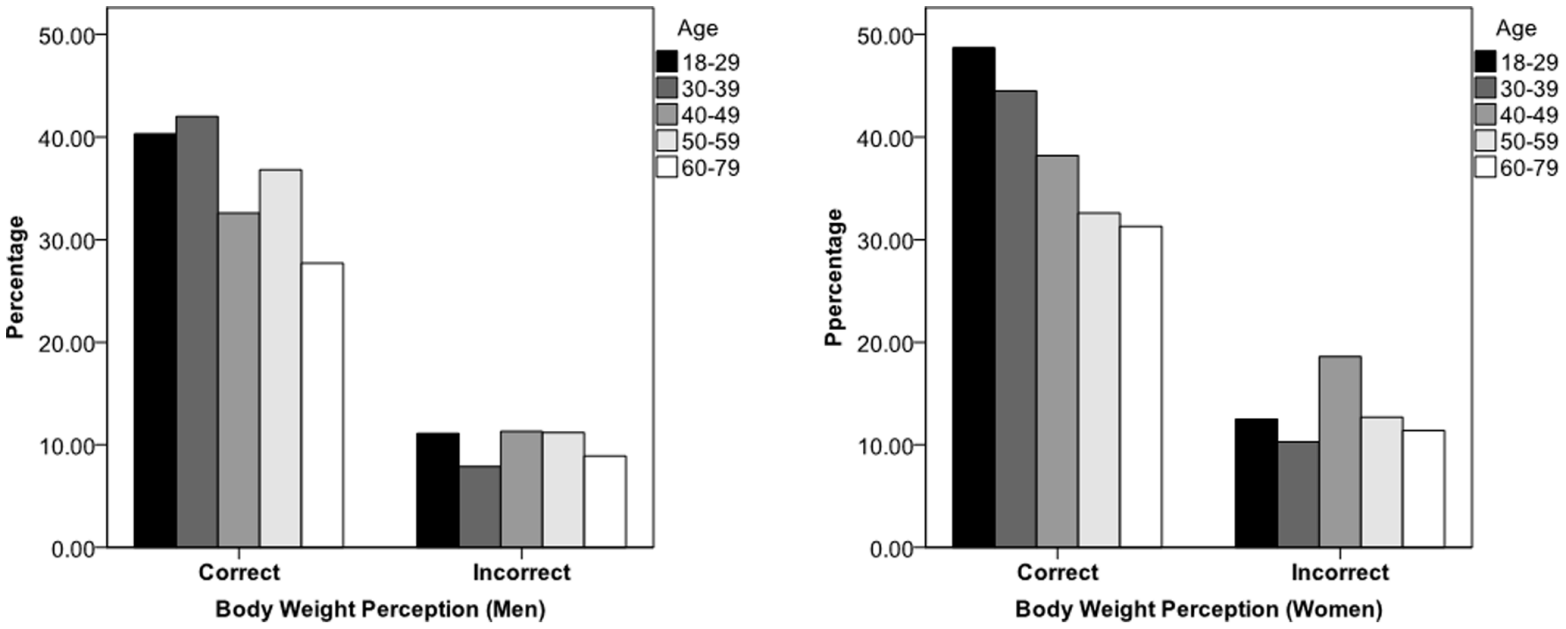
Percentage of taking action to lose weight by Body figure perception among those overweight or obese.

Those overweight or obese are recommended to lose weight to reduce risks of diseases; however, only 26.1% took action to lose weight. As shown in **Figure 1**, this percentage was very low among those overweight and obese participants with an incorrect perception of their body weight (10.2% in men, 13.3% in women). Even among those with a correct perception, the corresponding percentage was still low (35.9% in men, 37.3% in women) and decreased with age.

### Weight loss practice

As shown in **Table 5**, among all the participants taking action to lose weight, the top two practices were to reduce the amount of food intake and exercise. Most people chose dietary adjustment (including: to reduce the amount of food intake, low-fat diet, and low-calorie diet) and exercise; only a small portion of them adopted drug treatment and other practices. Interestingly, women were more likely to adopt “reduce the amount of food intake” and “drug treatment” and were less likely to choose “exercise” compared to men. Subgroup analyses among those overweight or obese showed similar results.

**Table 5:** Percentage of specific weight loss practice by sex among persons taking action to lose weight.

	Among all persons‡			Among those overweight‡			Among those obese‡		
	Men	Women	All	Men	Women	All	Men	Women	All
Reduce the amount of food intake	60.3*	71.4	68.0	61.9†	71.8	68.3	63.5	68.8	66.6
Low-fat diet	17.7	20.8	19.9	21.6	19.6	20.3	13.1	19.0	16.6
Low-calorie diet	23.5	25.6	25.0	20.5	25.7	22.4	17.2	24.9	21.7
Exercise	58.8*	50.2	52.9	61.0†	52.5	55.5	62.1	53.2	56.9
Use drug treatment	0.9*	7.2	5.3	0.5*	8.2	5.5	2.1	3.9	3.1
Others	0.2	0.7	0.5	0.0	0.7	0.5	0.7	1.5	1.1

*P<0.01: men vs. women.

† *P*<0.05: men vs. women.

‡ “all persons” referred to persons taking action to lose weight; “those overweight” and “those obese” referred to those overweight or obese persons taking action to lose weight.

## Discussion

This study showed that the prevalence of overweight and obesity was high in the general adult population in Beijing, as more than half of them were either overweight or obese. Incorrect body weight perception existed in nearly half of this population. Less than 30% of those overweight or obese were taking action to lose weight and those who considered themselves “fat” were most likely to take action. The most popular self-reported weight loss practices were to reduce the amount of food intake and exercise.

The prevalence of overweight and obesity in China has increased rapidly in the past decades. Using data collected in nine provinces, a recent study indicated that the prevalence of overweight and obesity increased from 36.1% to 40.1% in urban area and from 26.7% to 38.3% in rural area during 2000–2009 [1]. The recent 2010–2011 China National and Health Survey showed that the prevalence of overweight and obesity in urban residents were 32.4% and 13.2% [17]. Our study demonstrated that the prevalence in Beijing was higher than the national mean level and catching up to that in the developed countries [5], [18]. The increase in this prevalence has been mainly attributed to high-fat/calorie diet, sedentary lifestyle and lack of physical activity. The traditional Chinese diet is well-balanced, rich in fiber and low in saturated fats but now is shifting towards a diet with high fat, high energy density and low dietary fiber [19]. In 2002, daily mean percentage of calories for total fat was 35.0% in Chinese urban areas [20], which was higher than the WHO recommendation level (30%). It was reported that more Chinese urban residents (7.4%) than rural residents (2.6%) adopted a sedentary lifestyle in 2002 [21]. This percentage was even higher in large cities. For example, Yang et al. found that 20.5% of the Shanghai residents adopted a sedentary lifestyle [22]. Furthermore, average weekly physical activity among adults in China fell by 32% between 1991 and 2006 [23]. The urbanization factors were associated with 57% of the decline in total physical activity for men and 40% of the decline for women.

Overweight and obesity was more prevalent in men compared with women in all age groups except the age group of 60–79. The prevalence of overweight increased with age in both men and women, while the upward age trend in the prevalence of obesity was only seen in women. The observed gender and age differences were similar to the previous studies [1], [24]. Apart from age, the rapid hormone changes during menopausal transition may also contribute to the BMI and fat distribution change in middle age and older women [25], [26]. Interestingly, we found that both the prevalence of overweight and obesity varied by education level in women but not in men. This difference remained after adjustment for age. Similar findings were found in another study [27]. However, the relationship of education and obesity was not consistent [28]. Possible explanations is that higher educated women in Beijing were more aware of the health risks of excess body fat and had a greater pressure to have a socially ideal figure [7]. Given the adverse health consequences of overweight and obesity and their high prevalence, efforts from the national to the individual level are needed to slow this epidemic.

However, a large proportion of this population misclassified their body weight. Women were more likely to overestimate their body weight compared with men, especially young women. One third of normal weight women considered themselves “fat”, which could be partly explained by the fact that there is a prevailing belief in China that women should be slim and petite [7]. Even more concerning is that many of those overweight and obese were unaware of their weight problem. More than 40% of the overweight participants underestimated their body weight and fatness. It has been raised that overweight individuals may be more likely to self-perceived as “normal” in a setting where overweight and obesity are common [29]. This might be in part due to the lack of health knowledge on body weight, as higher proportion of under perception were observed in those with lower education level. Gao et al. also found that education level was positively related to correct perception of body weight [30]. Furthermore, a belief of body fatness representing prosperity and health is still widespread [7], especially in elderly, which may also partially account for the fact that older people underestimated their body weight to a greater extent than did younger people.

It has been demonstrated that weight perception is a motivating factor for weight control behaviors [13]. Consistent with this viewpoint, our study showed that self-perception of being fat was highly correlated with taking action to lose weight in both men and women. Among overweight and obesity individuals, who needed to lose weight for health interest, near 40% of those self-perceiving as “fat” (correct perception) took action to lose weight; however, the corresponding percentage in those with an incorrect weight perception was as low as 10.2% in men and 13.3% in women. Improving weight perception appropriateness through widespread health education programs thus is of health significance. Even among those overweight and obese individuals with a correct weight perception, more than 60% did not take action to lose weight. We found these people had a lower education level and were younger than their counterparts. This fact may be partly attributed to lacking information of the health hazards of overweight/obesity and responding to self-threatening information in a defensive manner. Studies had showed that self-affirmation could reduce this defensiveness [31]–[33]. Also, the general public perception is that achieving long-term weight reduction is difficult [34], which reduces people's desire to initiate weight lose practice. More studies are needed to explore the reasons for this dilemma.

Other factors associated with taking action to lose weight including sex, age, education level and BMI. Of particular concern was that men, elderly, and those with lower education level were less likely to take action although overweight and obesity were more prevalent in these subgroups. These findings were consistent with other studies [14], [35]. Specific attention should be given to these subgroups and more understanding of health and body perceptions of this population is needed. Similar to other studies [9], we found that the percentage of taking action to lose weight was increased with BMI. However, there was only a weak though significant relationship between them after adjusted for other variables.

Various weight loss practices were reported by those took action to lose weight. The common strategies were to reduce the amount of food intake (68%) and exercise (52.9%), which was similar to Weiss's study reporting that 64.7% of those trying to lose weight adopted eating less food and 61.3% adopted exercise [35]. However, Weiss et al also found that less than one fourth combined caloric restriction with the higher levels of physical activity (300 or more minutes per week), which was recommended for weight loss in the 2005 US dietary guidelines [35]. In our study, it may also be the case that some persons reported using physical activity to lose weight did not have enough exercise. We found a gender differences in weight loss practices. Among those took action to lose weight, women were more likely to reduce the amount of food intake but less likely to exercise. Particularly, these women were 8 times more likely to use drug treatment than man although anti-obesity drugs are not encouraged as first line treatment. This may reflect the societal pressure toward thinness among women.

This study has several limitations. Firstly, more women than men were included in our study, which might lead to bias in the overall prevalence of overweight and obesity. However, all the analyses in our study were stratified by sex. We also reported the results for men and women separately. Secondly, the information of weight loss practice was based on self-report. Socially undesirable practices such as drug treatment or vomiting might be under reported. Thirdly, the Chinese BMI classification were used, therefore it is not convenient to compare our result with those in other countries directly although this classification is more applicable to Chinese. Finally, the cross-sectional design of this study precludes causal relationships to be identified.

## Conclusion

Overweight and obesity were highly prevalent in the general population in Beijing. Nearly half of this population did not have correct weight perception. Less than 30% of overweight or obese individuals took action to lose weight. Our results suggest that efforts from the national to individual level are needed to slow or control the overweight and obesity epidemic.

## Supporting Information

Table S1 **Prevalence of overweight and obesity by selected characteristics and the corresponding odds ratios in Beijing Adults aged 18–79 years, 2011^§^.** ^§^Body size was categorized according to the WHO criteria for defining overweight (BMI 25–29.9) and obesity (BMI≥30).
(DOC)
