# Supplementary material for: Prevalence of Overweight and Obesity and Weight Loss Practice among Beijing Adults, 2011
Source: PLoS One. 2014 Sep 16;9(9):e98744. doi: 10.1371/journal.pone.0098744 (PMC4165756; doi:10.1371/journal.pone.0098744)
Supplement: Table S1 — Prevalence of overweight and obesity by selected characteristics and the corresponding odds ratios in Beijing Adults aged 18–79 years, 2011§. §Body size was categorized according to the WHO criteria for defining overweight (BMI 25–29.9) and obesity (BMI≥30). (DOC) [file pone.0098744.s001.doc]

**Supplementary data for**

**Prevalence of Overweight and Obesity and Weight loss Practice among Beijing Adults, 2011**

Li Cai^1,4^, Xiaoyan Han^2^, Zhi Qi^2^, Zhe Li^2^, Yumei Zhang^1^, Peiyu Wang^3^, and Aiping Liu^3*^

^1^ Department of Nutrition and Food Hygiene, School of Public Health, Peking University Health Science Center, Beijing, China

^2^ Chaoyang District Centre for Disease Control and Prevention, Beijing, China

^3^ Department of Social Medicine and Health Education, School of Public Health, Peking University Health Science Center, Beijing, China

^4^ School of Public Health, Sun Yat-sen University, Guangzhou, China

**Table S1 Prevalence of overweight and obesity by selected characteristics and the corresponding odds ratios in Beijing Adults aged 18-79 years, 2011^§^**

|  | *n* (%) | | | |  | Odds Ratios^*^ | | | |
| --- | --- | --- | --- | --- | --- | --- | --- | --- | --- |
|  | Men | | Women | |  | Men | | Women | |
|  | Overweight | Obesity | Overweight | Obesity |  | Overweight | Obesity | Overweight | Obesity |
| **Total** | 1059 (41.3) ^†^ | 240 (9.4) ^‡^ | 1390 (34.0) | 321 (7.9) |  | 1.37 (1.23, 1.52) | 1.20 (1.01, 1.43) | 1.00 | 1.00 |
| **Age** |  |  |  |  |  |  |  |  |  |
| 18-29 | 105 (24.5) ^†^ | 45 (10.5) ^†^ | 86 (12.9) | 14 (2.1) |  | 1.00 | 1.00 | 1.00 | 1.00 |
| 30-39 | 165 (40.3) ^†^ | 49 (12.0) ^†^ | 178 (24.5) | 39 (5.4) |  | 2.11 (1.57, 2.83) | 1.15 (0.75, 1.77) | 2.09 (1.57, 2.77) | 2.39 (1.28, 4.46) |
| 40-49 | 245 (40.8) | 62 (10.3) | 390 (41.1) | 85 (9.0) |  | 2.11 (1.60, 2.77) | 0.97 (0.64, 1.46) | 4.22 (3.24, 5.51) | 3.53 (1.97, 6.33) |
| 50-59 | 290 (48.7) ^†^ | 42 (7.1) | 335 (40.3) | 77 (9.3) |  | 2.91 (2.21, 3.83) | 0.65 (0.42, 1.01) | 4.01 (3.05, 5.26) | 3.61 (2.00, 6.51) |
| 60-79 | 254 (48.0) | 42 (7.9) ^‡^ | 401 (43.8) | 106 (11.6) |  | 2.87 (2.17, 3.80) | 0.70 (0.45, 1.10) | 4.43 (3.37, 5.83) | 4.16 (2.32, 7.48) |
| *P* for trend | <0.001 | 0.015 | <0.001 | <0.001 |  |  |  |  |  |
| **Education** |  |  |  |  |  |  |  |  |  |
| < High school | 455 (41.9) | 111 (10.2) | 667 (41.5) | 186 (11.6) |  | 1.00 | 1.00 | 1.00 | 1.00 |
| High school | 371 (42.8) ^†^ | 68 (7.9) | 437 (33.8) | 89 (6.9) |  | 1.10 (0.91, 1.32) | 0.73 (0.53, 1.00) | 0.88 (0.75, 1.03) | 0.67 (0.51, 0.88) |
| ≥ College | 232 (38.2) ^†^ | 60 (9.9) ^†^ | 283 (24.1) | 45 (3.8) |  | 0.96 (0.78, 1.18) | 0.89 (0.64, 1.25) | 0.67 (0.56, 0.81) | 0.43 (0.30, 0.61) |
| *P* for trend | 0.200 | 0.587 | <0.001 | <0.001 |  |  |  |  |  |

^*^ Odds ratios were adjusted for sex age, and education level.

^†^ *P*<0.01: men vs. women. ^‡^ *P*<0.05: men vs. women.

^§^ Body size was categorized according to the WHO criteria for defining overweight (BMI 25-29.9) and obesity (BMI≥30).
